# Gut microbiota: a crucial player in the combat against tuberculosis

**DOI:** 10.3389/fimmu.2024.1442095

**Published:** 2024-10-22

**Authors:** Jie Lin, Dongli Chen, Yongen Yan, Jiang Pi, Junfa Xu, Lingming Chen, Biying Zheng

**Affiliations:** ^1^ Guangdong Provincial Key Laboratory of Medical Molecular Diagnostics, Guangdong Medical University, Dongguan, Guangdong, China; ^2^ Institute of Laboratory Medicine, School of Medical Technology, Guangdong Medical University, Dongguan, Guangdong, China; ^3^ The Marine Biomedical Research Institute, Guangdong Medical University, Zhanjiang, Guangdong, China

**Keywords:** dysbiosis, tuberculosis, gut microbiota, microecology, gut-lung axis

## Abstract

The mammalian gastrointestinal tract quickly becomes densely populated with foreign microorganisms shortly after birth, thereby establishing a lifelong presence of a microbial community. These commensal gut microbiota serve various functions, such as providing nutrients, processing ingested compounds, maintaining gut homeostasis, and shaping the intestinal structure in the host. Dysbiosis, which is characterized by an imbalance in the microbial community, is closely linked to numerous human ailments and has recently emerged as a key factor in health prognosis. Tuberculosis (TB), a highly contagious and potentially fatal disease, presents a pressing need for improved methods of prevention, diagnosis, and treatment strategies. Thus, we aim to explore the latest developments on how the host’s immune defenses, inflammatory responses, metabolic pathways, and nutritional status collectively impact the host’s susceptibility to or resilience against *Mycobacterium tuberculosis* infection. The review addresses how the fluctuations in the gut microbiota not only affect the equilibrium of these physiological processes but also indirectly influence the host’s capacity to resist *M. tuberculosis*. This work highlights the central role of the gut microbiota in the host–microbe interactions and provides novel insights for the advancement of preventative and therapeutic approaches against tuberculosis.

## 1 Introduction

Tuberculosis (TB) is a highly contagious disease caused by *Mycobacterium tuberculosis* (*Mtb*) (1). *Mtb* evades the host immune system and develops drug resistance, complicating treatment. While the Bacillus Calmette-Guérin (BCG) is widely used to protect against TB, it provides limited protection in adults against pulmonary TB (2). Furthermore, mutations in genes that regulate *Mtb* growth and division during drug exposure can lead to antibiotic resistance, making TB difficult to treat and increasing susceptibility to lung inflammation (3, 4). Therefore, currently, preventing and treating *Mtb* infections remains a significant challenge in patients with TB.

The gut microbiota, which consists of microbes living in our digestive tract, plays a crucial role in various bodily functions, such as metabolism, immunity, digestion, and other aspects of the body (5). The gut microbiota influences host immunity and the penetrability and growth rate of the mucosal mucus layer, thereby becoming the first line of defense against foreign antigens (6, 7). Recent research has shown that imbalances in the gut microbiota, known as dysbiosis, can impact immune responses and lung infections (8–12). Several studies have reported that gut dysbiosis in extraintestinal diseases demonstrates the potential of indigenous gut bacteria to influence cross-organ diseases, particularly TB (3, 5, 8–13). It has been suggested that the gut microbiota communicates with the lungs, regulating lung function, managing inflammation, and providing protection against *Mtb* infection.

Given the link between gut microbiota and TB, it is crucial to understand this connection. This timely review discusses recent research on the involvement of gut microbiota in controlling or managing TB. We explore the role of the microbiota in the immune response, genetic regulation, and inflammation, all of which contribute to TB development. We also review practical implications from clinical studies and the potential relationship between TB and gut microbes. Furthermore, we examine the factors affecting gastrointestinal microbiota diversity, including drug usage, dietary patterns, host genetic variations, and microbial transplantation. Since gut microbiota plays a key role in maintaining microecological homeostasis, it could be a promising novel therapeutic target for TB treatment.

## 2 Gut microbiota–lung interaction

Symbiotic bacteria play an essential role in maintaining optimal lung immune function. A recent study showed that when dams were treated with polymyxin B during gestation, the gut microbiota of the pups altered (14). This treatment also induced a possible immune deficiency in these pups, as indicated by fewer splenic CD4^+^ T cells producing interleukin-2 after BCG vaccination, leading to their increased susceptibility to *Mtb* invasion (14). Additionally, an imbalance in the gut microbiota and mucus can cause pathological bacterial translocation, which can easily affect the extraintestinal organs (15, 16). The disruption in mucus secretion and stratification leads to “leaky gut” conditions, where intestinal permeability is increased, allowing extensive lipopolysaccharides (LPS) produced by Gram-negative bacteria (during dysbiosis) to enter the circulation (15). This process enhances lung inflammation by raising the levels of inflammatory cytokines such as IL-1β, IL-6, and TNF-α (17). A reduction in key mucins and tight junctions enhances lymphatic circulation, highlighting the enhanced crosstalk between the gut microbiota and the lungs.

The gut microbiota is recognized as a stimulator of the host immune cell repertoire. Gut bacteria can support the growth of innate immune cells by promoting hematopoiesis and regulating metabolism (18, 19). Dendritic cells (DCs) play a key role in bridging innate and adaptive immunity and primarily respond to antigen presentation among natural immune cells. The gut microbiota helps maintain the function of lung DCs, as it enables robust recognition and defense against *Mtb* infections (20–22). Through the secretion of microbial metabolites, the induction of inflammatory responses, and the regulation of immune reactions, the influence of gut microbial activity extends to the lungs via circulation (23). This process affects how immune cells respond and how well they can fight off pathogenic respiratory bacteria.

Interactions among the gut, microbes, and lungs help maintain the microecological balance of the body to fight against *Mtb*. Short-chain fatty acids (SCFAs), produced by the gut microbiota, have been implicated in the construction of the intestinal barrier and respiratory immunity. For example, succinate, a precursor of SCFAs, promotes pulmonary alveolar macrophage polarization and alveolar epithelial cell apoptosis during intestinal ischemia–reperfusion lung injury (24). Thus, succinate helps defend against pathogens. Butyrate, a SCFA, promotes the polarization of M2 macrophages, which helps reduce ongoing *Mtb* infection (25, 26). Research indicates that when M2 macrophages are primed by butyrate, the expression of goblet cell marker genes, including mucin2 (MUC2) and SPDEF, significantly increases in these immune cells (25, 26). Importantly, this process results in increased amounts of mucus, demonstrating the promising effects of microbial metabolites in repairing the mucosal barrier and pathogen clearance. SCFAs produced by the microbiota also enable signal transduction, thereby preventing excessive inflammation and tissue damage during TB infection. This process involves the promotion and proliferation of innate lymphoid cells (e.g., ILC3) via the metabolite-sensing Ffar2 receptor, which increases the expression of alveolar macrophages and enhances pathogen-killing against *Mtb* infection (27, 28).

These findings suggest that targeting the gut microbiota likely enhances the host immunity and reduces susceptibility to disease. The gut microbiome impacts overall health, with gut–lung interactions highlighting the need for interdisciplinary teamwork across microbiology, immunology, and respirology. However, more research is needed to fully understand the regulatory mechanisms associated with microbial stimuli.

## 3 Gut microbiota in TB infection

In patients with active TB infection or latent TB infection, two prominent gut phyla*—Firmicutes* and *Bacteroidetes—*show correlation with inflammatory biomarkers like IL-1β and IL-4 (29). Specifically, patients with active TB show a lower *Firmicutes*-to-*Bacteroidetes* (F/B) ratio, indicating a potential connection between inflammation, gut microbes, and TB development (30). Moreover, *Mtb* infection disrupts microbial metabolic pathways, leading to an elevated likelihood of inflammation and worsening TB. Compared to healthy individuals, patients with TB exhibit decreased biosynthesis of amino acids and fatty acids (31, 32). Notably, nucleotide metabolism pathways are significantly more active in patients who are not on antibiotics (the antibiotic-free TB group), with the greatest fold changes in related genera, such as *Enterococcus*, *Clostridioides*, and *Rothia* (32). These metabolic changes indicate altered energy transformation in patients with TB, which may be restored by sustaining certain prebiotics.

During the inflammatory response, the microbial metabolite like SCFAs regulate signaling pathways and influence metabolic switching mechanisms. Upon stimulation by the *Mtb* antigen, SCFAs inhibit the production of IFN-γ and IL-17, which modifies the microenvironment of the lung microbiota (33). While proinflammatory cytokines such as IFN-γ and IL-17 normally help limit inflammation by activating immune cells and controlling the pathogen spread, their reduced levels during *Mtb* infection impair the early immune response against TB (34, 35). This observation is consistent with the increased abundance of SCFA-producing anaerobes (36). Increased levels of SCFAs are also associated with the activation of TB-induced regulatory T cells (Tregs), which impede *Mtb* invasion and reduce lung lesions and bacterial load, helping to prevent excessive inflammation and promote immune tolerance (37). Mechanistically, SCFAs interact with cell-surface receptors like G-protein-coupled receptor 43 (GPR43), profoundly affect the inflammatory response, and improve recovery from inflammation (38).

Accordingly, the inflammatory trajectory of a disease may be determined by the type and quantity of gut flora that confer great benefits. Notably, some studies have suggested that an increase in the relative abundance of the genus *Bacteroides* is the main cause of gut microbiota dysbiosis in patients with pulmonary TB (39, 40). High levels of *Bacteroidetes* are positively associated with polymorphic neutrophils in the blood of patients with active TB (29). For example, *Bacteroides fragilis*, a prominent species of the *Bacteroides* genus, and its capsular polysaccharide-A (CPS-A) were shown to inhibit pulmonary inflammation by promoting IL-10 production by CPS-A-responsive T cells and antigen-presenting cell-mediated CPS-A processing (41). Moreover, the administration of *B. fragilis* or polysaccharide (PSA) to germ-free mice can restore the Th1/Th2 cytokine balance by boosting the production of Th1 cytokines, including IFN-γ and IL-2 (42). Specific microorganisms that exhibit conspicuous alterations in inflammation can interfere with the disease progression.

*Anaerobes* are found in higher levels in the sputum and stool of patients with pulmonary TB and are correlated with the activation of proinflammatory immune pathways, such as interferon and Nur77 signaling, as well as inflammasome pathways (8). Certain pathways have shown a positive correlation with the presence of the genus *Anaerostipes* bacteria in the gut (43, 44). In recent years, there has been a growing interest in the role of *Akkermansia muciniphila* in inflammatory diseases. Metabolites originating from *A. muciniphila*, such as the tripeptide Arg-Lys-His, the outer protein Amuc_1100, and palmitoleic acid, have been shown to have positive effects on mice by regulating metabolism and cellular signaling (45–47). Furthermore, a decrease in the abundance of *A. muciniphila* in the gut microbiota of patients with active TB is negatively correlated with low-grade inflammation, indicating the involvement of regulatory T cells and Toll-like receptors in mediating the immune responses (47, 48).

TB recurrence is often linked to immune fatigue and the suppression of inflammation. Compared to healthy individuals, patients with TB specifically exhibit reductions in beneficial bacterial genera, such as *A. muciniphila* and *Bifidobacterium*, and an increase in potentially harmful bacteria within the *Proteobacteria* and *Actinobacteria* phyla in the gut (32, 40, 47). However, changes in microbial populations can vary based on disease states and dietary habits, and changes in the abundance of microbial populations are not necessarily absolute (49, 50).

**Figure 1:**
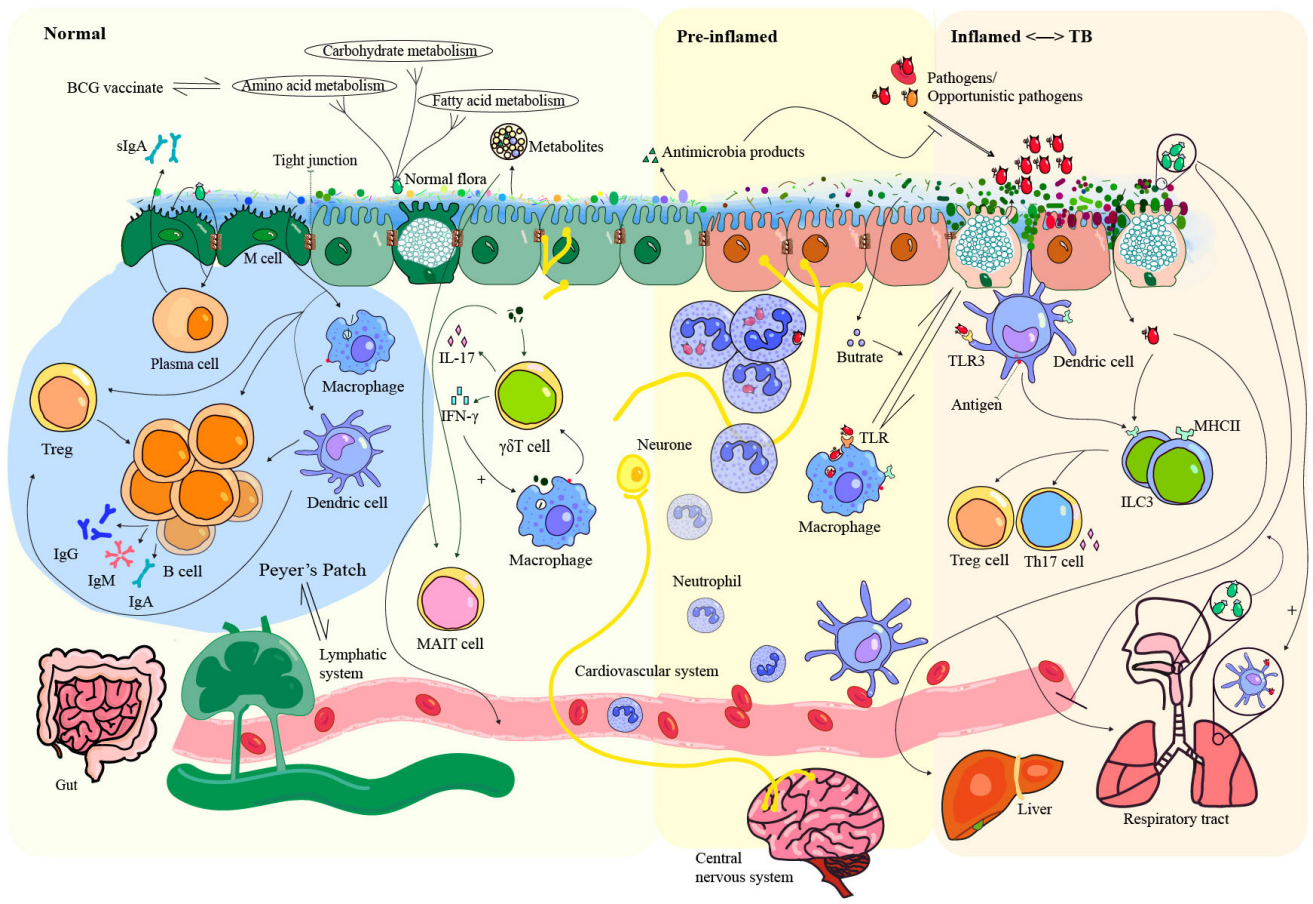
Interaction between microbiota, pathogens, and host immunity in health and TB-related inflammation. The gut microbiota plays a critical role in the construction of the mucosal barrier by interacting with immune cells, contributing to colonization resistance, and secreting metabolites that help maintain homeostasis. Close contact between luminal microbiota and epithelial cells triggers an immune response that can lead to inflammation and increase the likelihood of attack by opportunistic pathogens. Partial immune evasion by these pathogens can allow them to spread to organs such as the liver, lungs, and brain via the bloodstream or lymphatic circulation. Importantly, the commensal microbiota in both the gut and respiratory tract helps prevent lung infections by stimulating lung dendritic cells. The gut microbiota exhibits great potential as a reflector and modulator of TB. TLR, Toll-like receptors; Treg, regulatory T cell; MHCII, major compatibility complex II; ILC3, group 3 innate lymphoid cell; MAIT cell, mucosal-associated invariant T cell.

Overall, gut dysbiosis in patients with TB represents a complex stalemate between *Mtb* invasion, immune activation, and gut microbiota (**Figure 1**). Gut microbiota and their metabolites affect different immune cells, mediating pro- or anti-inflammatory signaling, disease development, and recovery. They play a significant role in the occurrence and development of diseases, not only influencing the diagnosis and treatment of diseases but also being capable of predicting clinical outcomes and prognosis and providing novel immunotherapies. Further studies on the types of metabolites produced by the gut microbiota and the underlying mechanisms could clarify the role of the microbiota–immune axis in TB management.

## 4 Gut microbiota in latent TB and drug-resistant TB

Latent tuberculosis infection (LTBI) is asymptomatic and poses a challenge for the timely detection of this condition. A healthy gut microbiota supports immunity and nutrient absorption, aiding the body to defend against TB. It also undergoes functional changes at different stages of the disease, which can influence TB pathogenesis. Dysregulation of the gut microbiota might be an early immune response to *Mtb* invasion, which likely occurs before inflammation begins. In a related study, patients with latent TB showed high levels of *Bacteroidetes* in their gut and an increased number of polymorphic neutrophils in their blood, though without inflammation (29). In addition, the abundance of *Coriobacteriaceae* appears to be associated with CD4^+^ T cells and the expression of IFN-γ (29). Therefore, a strategic approach for predicting the development of TB using microbiota with innate immunity-modulating properties holds promise. However, some studies reported no significant differences in gut microbiota composition between individuals with LTBI and healthy controls (49). Thus, further research is needed to understand the potential role of the gut microbiota in diagnosing latent TB.

Interestingly, changes in the gut microbiota have been reported by certain studies prior to the diagnosis of latent TB. Distinctive gut microbial signatures have been observed not only between LTBI patients and healthy individuals but also between individuals exposed to patients with TB and those who are not (49, 51). This study highlights the sensitivity of the gut microbiota to pathogen exposure and its potential for the early diagnosis of TB.

Interestingly, the composition and abundance of the gut microbiota appear to be an indicator of tuberculosis drug resistance. Multidrug-resistant TB (MDR-TB) is challenging to treat. A study conducted in patients with pre-extensive drug-resistant TB revealed higher levels of *Enterobacteriales*, *Bifidobacteriales*, *Verrucomicrobiales*, and *Lactobacillales* in the coliform flora (52). This microbial imbalance in the gut microbiome was linked to elevated *de-novo* fatty acid synthesis in patients with MDR-TB, with *Bacteroides* displaying significant correlations. Both drug resistance and immune evasion mechanisms contribute to the formation of biofilms by *Mtb*, contributing to its survival (53). Therefore, identifying the key factors that contribute to the increased susceptibility to multidrug-resistant TB, such as alterations in the gut microbiota and metabolic changes resulting from long-term antibiotic use, can help in predicting and treating this form of TB. The gut microbiota may affect the absorption, distribution, metabolism, and excretion of anti-TB drugs, thereby influencing the drug efficacy. By adjusting the composition of gut bacteria, theoretically, it is possible to change the intestinal microbial ecosystem, impacting the growth and reproduction of *Mtb* and even enhancing the drug activity against the bacteria.

## 5 Gut microbiota in TB control and prevention

### 5.1 Development of the TB vaccine

The gut microbiota significantly affects vaccine efficacy and plays a crucial role in preventing TB infections and fostering the development of a robust immune memory. BCG, a TB vaccine commonly employed in mass inoculations, offers cross-immunoprotection against *Mtb* infections in humans (54). However, its protective effect is limited in adults. A recent study found that the live BCG vaccine upregulated the expression of MHCII, TLR2, and genes involved in antigen presentation and processing in lung alveolar macrophages, compared with phosphate-buffered saline and heat-inactivated BCG (12). By spreading mycobacteria to proximal gut sites, parenteral administration of the BCG vaccine induces decreased microbial diversity, significant enrichment of *Lactobacillaceae*, irregular distribution of tight junction proteins, elevated levels of butyrate, and the development of mild colitis (12). Transplanting BCG-conditioned microbiota into naive mice elevated MHC II, IL-6, and TNF production in airway alveolar macrophages and triggered anti-TB-trained immunity, with similar effects observed in lung tissue (12). These findings shed light on the potential link between the vaccine response, trained immunity, and gut microbiota, providing valuable insights into the development of robust immune memory for future vaccine design.

Gut dysbacteriosis can reduce the efficacy of the BCG vaccines by inhibiting the function of *Mtb*-specific T cells, resulting in suboptimal pathogen clearance (55). However, trained immunity has valuable heterologous effects in TB vaccines (11). Reprofiling the gut microbiota can improve the trained and specific immune responses following vaccination. The gut microbiota and their metabolites can train cycling monocytes and modify the bactericidal function of alveolar macrophages, thereby affecting *Mtb* invasion (56). Both specific immunity and SCFAs produced by certain genera, such as *Eggerthella lenta* and *Roseburia*, are involved in immune regulation (57). For example, amplification of *Roseburia* levels has been shown to reduce the cytokine response and modify the phenylalanine pathway after BCG vaccination (57). These findings have established a direct link between BCG-altered microbiota and the activation of memory immune cells.

Immune cells exhibit the ability to respond to microbiota, suggesting that gut microbiota could help develop TB vaccines with enhanced immunogenicity (58). Identifying and cultivating the microbial species that can mediate and facilitate the interactions between innate and adaptive immune responses is crucial. Through their interaction with particular immune cells, certain gut microbiota help build a robust immune memory, which plays a key role in protection against TB.

### 5.2 How to prevent TB relapse

Research on gut microbiota in patients with recurrent tuberculosis is quite limited. One study demonstrated that patients with relapsing TB have higher gut microbiota diversity compared to that of healthy controls, distinguishing them from new-onset cases (40). Reduced abundance of *Bacteroidetes* and increased abundances of *Actinobacteria* and *Proteobacteria* are strongly associated with TB recurrence. To gain better insights into the potential use of microbiota in predicting TB recurrence, future studies should include larger sample sizes and exclude non-major factors.

**Figure 2:**
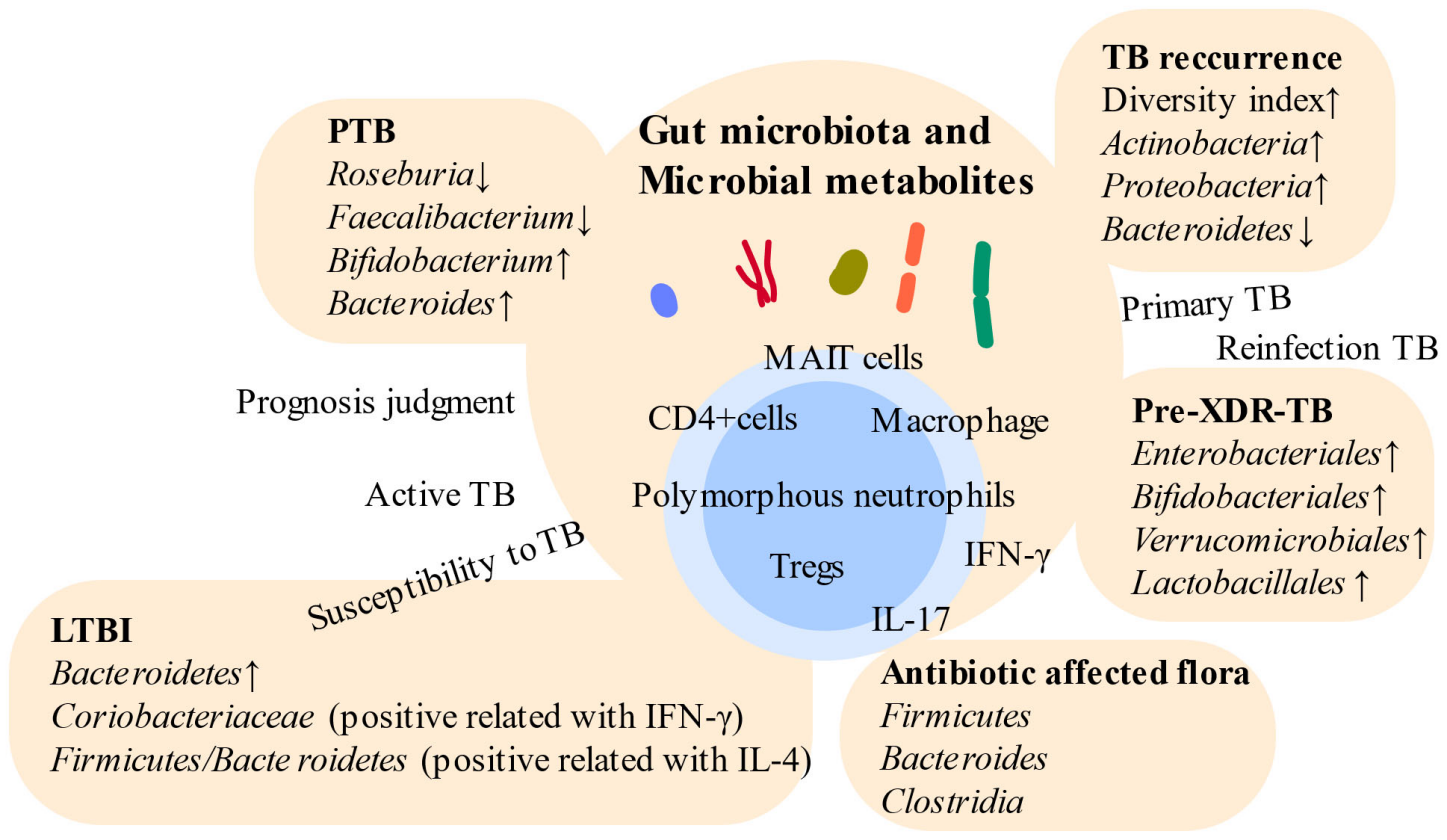
Relationships between gut microbiota and distinct TB. Gut microbiota vary across different stages of TB. Intestinal microbes become potential targets for indicating different states of TB and boosting immunity. However, high individual variation poses challenges in utilizing gut microbiota-based approaches for the diagnosis of TB. PTB, pulmonary tuberculosis; LTBI, latent tuberculosis infection; Pre-XDR-TB, pre-extensive drug-resistant tuberculosis; Tregs, regulatory T cells.

Dysbiosis of the gut microbiota caused by antibiotic treatment does not resolve after disease resolution, leading to persistent symptoms and an increased likelihood of TB recurrence (10, 14, 59). Gut microbiota may significantly affect the occurrence and development of TB (49). Consequently, there is a growing focus on studying changes in the microbiota at different stages of disease progression. The influence of various factors such as geography, dietary habits, age, constitution, and coexisting diseases on the gut microbiota has been investigated through comprehensive microbiota assessments conducted across diverse populations (60–62). Additionally, long-term aggressive regimens have proven effective in reducing the risk of recurrence in patients with MDR-TB (63). Maintaining the homeostasis of gut microorganisms has positive implications for both host prognosis and the anticipation of the recurrence of *Mtb* infection. TB-related microbiota are shown in **Figure 2**.

## 6 Considerations in the study of gut microecology in TB

### 6.1 Appropriate modeling

Selection of an appropriate model for studying the gut microbiota is crucial in TB research. The differences observed in the gut microbiota status between pulmonary and extrapulmonary tuberculosis, along with the severe pulmonary injury observed in *Helicobacter hepaticus*-colonized *Mtb*-infected mouse models, emphasize the importance of investigating multiple body sites and the varied outcomes associated with different commensal bacteria (64, 65). Notably, compared to pulmonary tuberculosis (PTB), extrapulmonary tuberculosis (EPTB) shows a greater association with drug resistance, underscoring the importance of examining gut microbiota in both forms of tuberculosis to elucidate the potential mechanisms underlying drug resistance (64).

Combined disease models may be helpful in revealing the TB characteristics of gut microecology. Typically, individuals who are highly susceptible to tuberculosis often have concurrent underlying health conditions. For example, diabetes mellitus is a critical risk factor for TB (51). Studies involving *Mtb*-infected mice with or without type 2 diabetes (T2D) have revealed distinct clusters in the gut microbiome, indicating the influence of metabolic disorders on the gut microbiota and their impact on respiratory disease susceptibility (66). Comorbid mouse models may provide insights into immune parameters, the metabolome, and the microbiome, thereby illuminating the complexities of the gut–lung axis. Further research is required to determine the validity and reliability of the gut microbiota as an indicator of host differences in various diseases and conditions.

### 6.2 Genetic calculations

Host genetics has been found to affect TB infection and lead to differences in microbiota characteristics (34). If strong correlations can be clearly established between host gene expression and the gut microbiota and between the genetic composition of the microbiota and desirable host traits, this could facilitate selective breeding for an optimum microbiome.

**Figure 3:**
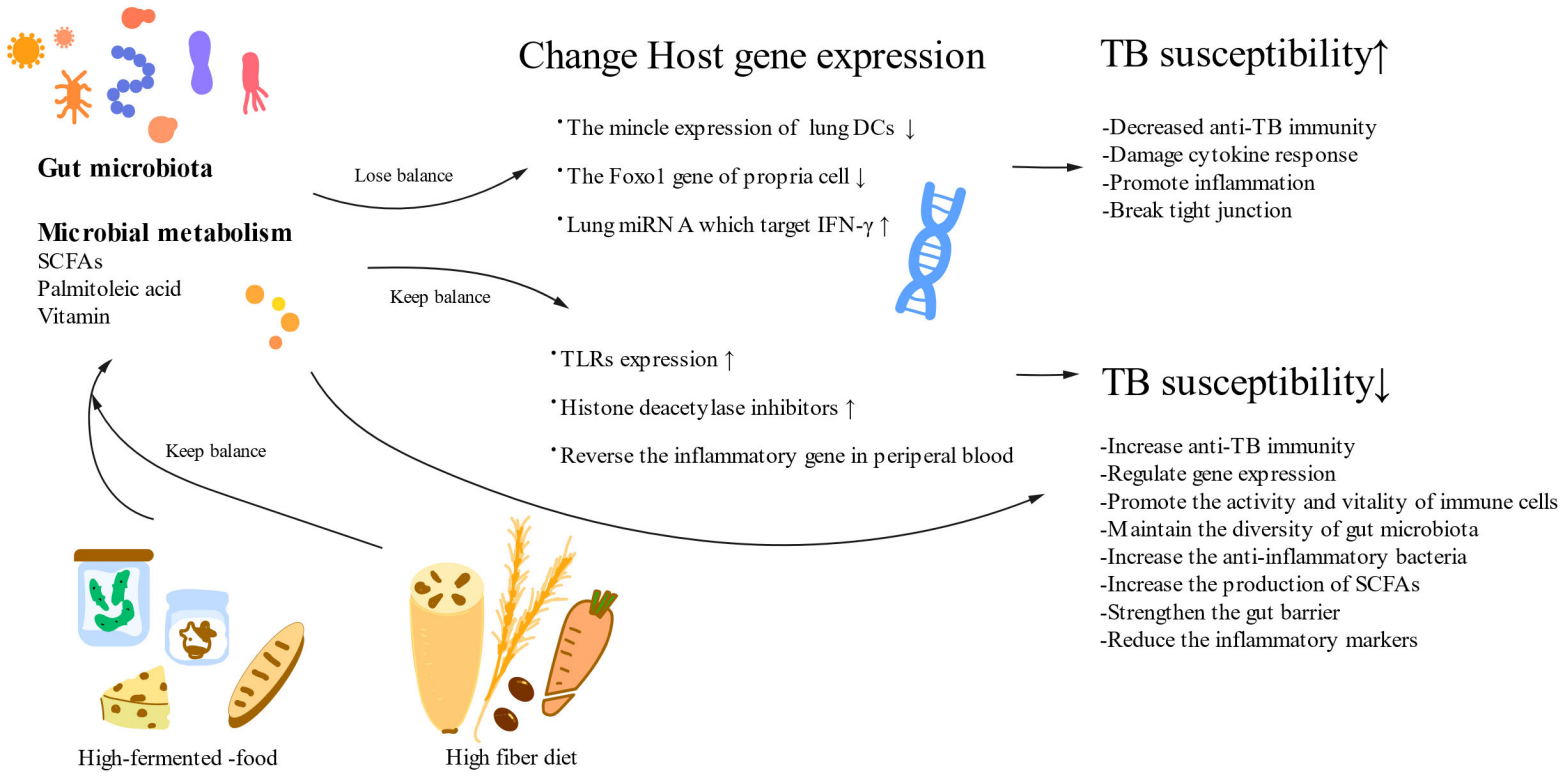
A healthy diet and normal gut microorganisms reduce TB susceptibility. A high-fermented food and a high-fiber diet can positively impact the gut microbiota and its metabolism, which can influence TB susceptibility through their effects on host genes. Gut microbiota changes anti-TB immunity by influencing host gene expression. SCFAs, short-chain fatty acids; DCs, dendric cells; TLRs, Toll-like receptors.

Gut microbes can influence genetic modifications by interacting with the genetic machinery of host cells (**Figure 3**). Disruption of the microbiota and its metabolites, especially SCFAs, may disrupt tight junctions and lead to a deficiency of Foxo1 in propria cells (67). SCFAs are involved in the regulation of gene expression, promotion of immune cell survival, and phagocytic activity enhancement, partly by inhibiting histone deacetylases (HDACs) (68). Among these metabolites, β-hydroxybutyrate, an inhibitor of HDACs, promotes intestinal cell differentiation and supports gut homeostasis by inhibiting mTOR signaling (69). SCFAs transmit signals into cells via G-protein-coupled receptors (GPCRs) and through transporters such as monocarboxylate transporter 1 (MCT1) and sodium-coupled monocarboxylate transporter 1 (SMCT1) (70). These receptors and transporters are highly expressed in colonocytes, but their potential involvement in regulating TB regulation requires further research.

From a broader perspective, gut microbiota are involved in strengthening respiratory defenses against pathogens by activating antigen recognition. Specific bacteria can stimulate Nod-like receptors in the upper airway and gut, defending against lung infection via the activation of Nod2 and granulocyte-macrophage colony-stimulating factor (GM-CSF) (71). Mechanistically, the NOD2 gene encodes a host sensor of a bacterial cell wall component called muramyl dipeptide (MDP), which is positively associated with CD103^+^ DCs in the gut lamina propria and stimulates GM-CSF production (72). Additionally, certain gut microbiota can induce the expression of Toll-like receptors to recognize *Mtb* cell wall components and initiate innate immune responses (73). *Mtb* stimulates the expression of the Irg1 gene in macrophages either by activating the STING pathway to produce type I IFN through the release of *Mtb* products or by inducing signaling dependent on TLR-2, MyD88, and NF-κB. This finding implies the potential utility of probiotics in increasing self-resistance to tuberculosis through the gut–lung axis and their promising role as therapeutic agents for intervention.

MicroRNAs play crucial regulatory roles in cellular functions and are influenced by the gut microbiome. The burden of bacilli and pathological injury can be reduced by IRF7-mediated miRNA-31, which upregulates positive genes in the lungs (74). Additionally, both gut dysbiosis and *M. tuberculosis* infection upregulate the expression of lung miR-21 (75). Specifically, the expression of miR-21-3p has been found to impair anti-TB immunity by targeting IFN-γ mRNA and downregulating the levels of IFN-γ, as indicated by the commensal flora (75). A recent study identified *B. fragilis* as a direct regulator of protective immunity against TB through modulation of lncRNA-CGB (3). lncRNA-CGB functions as a negative regulator of H3K27 trimethylation (H3K27me3) epigenetic programming, ultimately enhancing IFN-γ expression by interacting with EZH2, thereby providing anti-TB immunity (3). Consequently, exploring the inhibition of such miRNAs by the commensal microbiota is a promising therapeutic approach for enhancing host protective immunity against TB. Actually, miRNA and gut microbiota interact with each other. Gut microbiota intervention may offer another way to overcome the technical challenges faced by miRNA therapy, such as low delivery efficiency and instability in the body.

Distinct profiles of the gut microbiota are linked to inherent TB susceptibility, which is driven by host genetics. Risk alleles and age-at-onset play crucial roles in the progression of PTB. Genetic variations in humans, such as single nucleotide polymorphisms (SNPs), are correlated with the susceptibility to PTB (76). Genetic changes alter gut-colonizing microbes. For instance, in *Th2* gene knockout mice, alterations in the structure of the gut microbiota have been observed, coexisting with enhanced fat metabolism through the increased expression of uncoupling protein 1 (UCP1) and GPRs (77). In addition, the Ifnar1 rs2257167 G allele in the host is associated with an increased susceptibility to TB, which is characterized by a decreased abundance of *A. muciniphila* in patients with active TB (47). Understanding the mechanisms underlying individual variations in immunity and the gut microbiota may help to evaluate TB susceptibility.

### 6.3 Dietary intake

Dietary adjustments have the potential to induce alterations in the gut microbiota, turning the gut into a bioreactor (78). The gut microbiota are influenced by long-term dietary habits, and they can rapidly respond to changes in diet, exhibiting unique communities related to metabolism and composition (79). Clinical trials have shown that the introduction of microbially driven complementary food and low-calorie Mediterranean and vegetarian diets can cause substantial changes in the composition of the gut microbiota at the genus level and affect various biochemical parameters (80, 81). Food has a positive impact on plasma proteomic biomarkers associated with growth, development, and immune function (81).

Food combinations are linked to the gut–lung axis via microbial metabolism. The expression of the aryl hydrocarbon receptor (AhR) in pulmonary endothelial cells is induced by foodborne ligands, such as indole and tryptophan (82). AhR is a ligand-activated transcription factor that maintains the stability of the pulmonary barrier and eliminates infections. However, a high-fat diet depletes tryptamine and indole-3-acetate levels (83). Metabolism of these metabolites is dependent upon the microbiota and may reduce the levels of proinflammatory cytokines induced by LPS in macrophages in an AhR-dependent manner (83). Dietary interventions hold great promise for the treatment of metabolic diseases. Moreover, infectious diseases commonly coexist with metabolic abnormalities, such as decreased levels of SCFAs, amino acids, and fatty acids, in patients with TB (32). These studies further emphasize the potential influence of diet on health and its possible interactions with the gut microbiota.

In addition, research suggests that the gut microbiota can either activate or deactivate host genes in response to dietary stimuli (**Figure 3**) (84). The establishment of immune tolerance to food antigens has been linked to the location, diversity, and metabolic functions of the gut microbiota (85). The ingestion of highly fermented foods enhances gut microbial diversity and reduces inflammatory markers, thereby offering benefits in the context of TB treatment (86). In summary, understanding the interplay between dietary patterns, gut microbiota equilibrium, and disease is vital for manipulating the gut microecology through dietary interventions, ultimately offering potential therapeutic benefits for TB.

### 6.4 Fungi and viruses

In addition to gut bacteria, the role of fungi and viruses in the gut in relation to tuberculosis is also worthy of in-depth investigation. Fungi provide several benefits to the body, including anti-inflammatory effects, barrier construction, and modulation of the gut microbiota composition (87). Yeast can be degraded by certain bacteria to release mannans, which helps to prevent infections and reduce the occurrence of immune diseases (88). A previous study revealed that the diminished network after anti-TB treatment may be rebuilt by the enrichment of fungal nodes (89). Implementing adjunctive therapies that focus on curbing fungal proliferation and fostering the growth of beneficial bacteria after anti-TB therapy is crucial for accelerating the recovery of patients with TB.

Host–bacterial–fungal interactions are strongly associated with inflammation. An intriguing study revealed the anti-inflammatory properties of sophorolipid, a glucolipid compound synthesized by yeast species such as *Candida bombicola*, competitively bind to TLR4/MD-2, outcompeting LPS and inhibiting IKKβ activation via hydrogen bonding and hydrophobic interactions (90). This mechanism effectively suppresses inflammatory signaling. Another yeast species, *Candida albicans*, secretes a peptide toxin called candidalysin during its transition to a pathogenic state, reflecting aggravated intestinal inflammation through IL-1β-dependent mechanisms (91). However, the fecal bacterial dysbiosis and local inflammation induced by *Candida* may be attenuated by certain bacteria, such as *Lactobacillus rhamnosus* L34 (92). In summary, fungi regulate inflammation and interact with bacteria in the gut, thereby participating in disease management.

Viruses are also involved in intestinal homeostasis. Antigen-presenting cells recognize the cytosolic RNA of commensal viruses via the MAVS–IRF1–IL-15 axis to maintain the balance of intestinal intraepithelial lymphocytes (IELs) (93). Additionally, both antiviral treatments and deficiency of RIG-I—a receptor for cytosolic viral RNA—alter gut bacteria in mice (93). Membrane vesicles derived from the gut microbiota can deliver DNA into distal host cells via circulation and prime antiviral immunity by activating cGAS-STING (94). The interactions among viruses, bacteria, and the immune system are worth exploring by researchers in terms of the role of commensal viruses in infectious diseases.

Intestinal phages target pathogens and reduce inflammation. Phages are abundant in the human gut; however, most remain uncultured (95). The oral administration of phages that target certain pathogens may potentially inhibit inflammation and disease exacerbation (96). Moreover, phage predation-induced alterations in the microbiota have a significant impact on the gut metabolome (97). By specifically targeting certain bacteria, phages not only control the growth of pathogens but also promote the proliferation of beneficial flora that are advantageous in combating TB, thereby offering a novel perspective for improving the intestinal microbiome of patients with TB.

Currently, the application of phages targets multiple aspects of TB infections, including vaccine development, TB diagnosis, and the detection of drug resistance (98, 99). Carrier-mediated phage delivery aids in the recognition of intracellular bacteria (100, 101). However, in the context of gut microbiome modulation, phages may exhibit several potential drawbacks such as excessive specificity, the potential spread of drug resistance, triggering of the host immune response, disruption of ecological balance, and unpredictability. In conclusion, under appropriate conditions and reasonable application, phages may also be beneficial regulatory tools for TB management.

## 7 Gut microbiota: blueprint for the fight against TB

### 7.1 The usage of antibiotics

Antibiotic treatment not only modulates inflammatory gene expression in the peripheral blood, but also disrupts the gut microbiome by reducing its biodiversity and altering the composition of the gut microbiota, leading to pronounced and sustained dysbiosis (34, 89). Anti-TB drug treatments affect various intestinal microorganisms, including those belonging to the phylum *Firmicutes* and the genus *Bacteroides*, particularly the class *Clostridia* among anaerobic *Firmicutes*, which are the major phyla disrupted by HRZE therapy, which consists of isoniazid, rifampicin, pyrazinamide, and ethambutol (39, 102). Conversely, extreme alterations in the microbiota caused by broad-spectrum antibiotics may directly or indirectly affect the efficacy of anti-TB drugs (103). A deficiency in the gut microbiota also disrupts host immunity and impacts the host’s response to antibiotics, including a decrease in inflammatory, IFN-α, and IFN-γ responses (102). After antibiotic cessation, the residual impact on microbiota can persist for up to 3 months, and a significant proportion of the altered microbial taxa is associated with modifications in immunological functions (104). In mice infected with *Mtb*, mucosal immune function is weakened by antibiotic treatment, as indicated by a reduction in MAIT cells (105). Notably, high susceptibility to severe infections can be induced by the broad immunological effects of antibiotics.

Antibiotic-induced dysbiosis appears to cause metabolic dysfunction. Exposure to antibiotics is associated with the depletion of microbes that produce SCFAs (32). Butyrate has been shown to influence the expression of anti-inflammatory and proinflammatory factors that regulate macrophage function in clearing *Mtb* (32). Specifically, the abundance of *Actinomyces*, which are involved in butyrate production through the modulation of its precursors such as acetate and succinate, decreased during the intensive and continuation phases of second-line anti-TB treatment (106). In addition, the abundances of certain *Prevotella* species and *Megamonas hypermegale*, which are associated with glucose metabolism and SCFA production, decreased. Broken early resistance in the lungs is commonly observed in *Mtb*-infected mice due to dysbiosis of the gut microbiota caused by broad-spectrum antibiotics (105). Adverse outcomes caused by dysregulated microbiota may contradict to the benefits of anti-TB treatment.

Inflammation recovery may be observed when the microecological damage caused by antibiotics is improved. In one study, the normalization of inflammation and clearance of *Mtb* were associated with increased numbers of cluster IV and XIVa *Clostrida*, along with reductions in *Bacilli* and *Proteobacteria*, which were triggered by antibiotic treatment (102). Recovery from dysbiosis caused by continuous drug treatment affects immune cell proliferation and autophagic processes, reflecting the outcomes in patients with TB. Moreover, although short-term antibiotic treatment may not significantly alter gut microbiota in all infections, its effects still require further consideration (13). The dynamic and long-term effects of antibiotics on gut microbiota require further study in a diverse and representative population.

### 7.2 Healthy lifestyle and dietary habits

**Figure 4:**
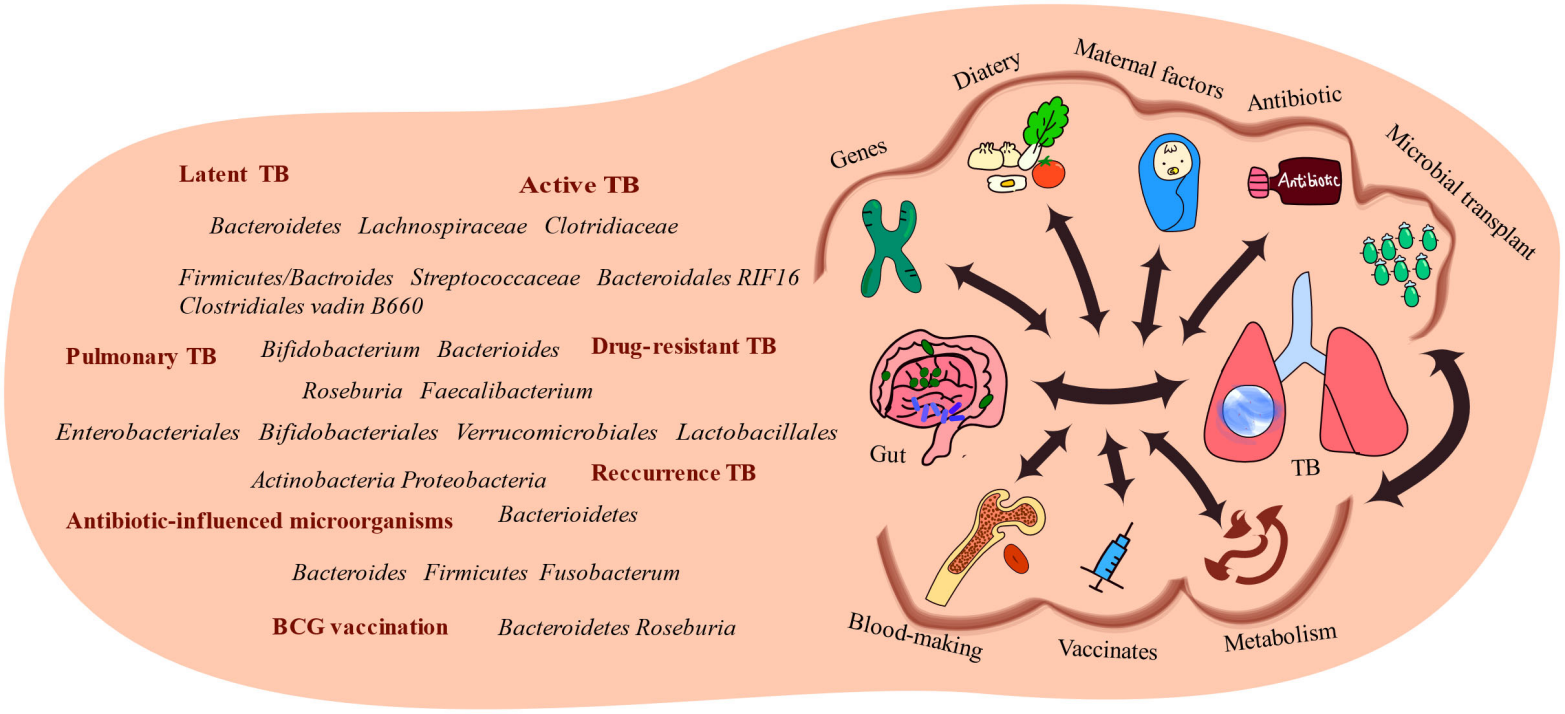
Several factors link the gut and the lungs. Alterations in gut microbiota have been observed in TB patients, highlighting the association between the gut microbiota and diseases. Several factors influence the interactions between gut microbiota and TB-infected organs, including genetic predispositions, dietary factors, material factors, antibiotic usage, and microbial transplantation. These factors influence the development and progression of TB.

Assessing species abundance is essential to determine whether a species plays a dominant role in the disease process. Notably, various factors, including but not limited to those mentioned previously, influence the modulation of the microbial community and the identification of specific disease-associated microorganisms within the ecosystem, highlighting the bidirectional relationship between habits and TB progression (**Figure 4**).

Smoking affects disease susceptibility and alters intestinal microbiota composition. Cigarette smoking is a major global health threat that contributes to a significant number of deaths annually. In mice, smoking and cessation disrupted the gut microbiota community due to the entry of cigarette smoke-related metabolites into the intestine, leading to dysbiosis (107, 108). The connection between gut microbiota and weight gain associated with smoking cessation has been demonstrated through gut microbial transplantation, and similar effects are seen following microbiome depletion by antibiotics. Dysbiosis induced by smoking is accompanied by increased bile acid metabolites as well as elevated oncogenic MAPK/ERK signaling, resulting in impaired gut barrier function (108). Conversely, specific gut microbes also influence the risk of cigarette smoking. A previous study suggested that a decreased abundance of *Actinobacteria* due to smoking may worsen smoking status, potentially exacerbating the reduction in *Actinobacteria* abundance, indicating a potential positive feedback effect (109). The negative effects of smoking not only directly impact the lungs but also regulate susceptibility to further diseases by influencing gut microbiota.

It is important to recognize the wide individual variations in gut microbiota composition due to lifestyle, diet, and environment to identify disease-associated microbiota. In a study by Ivan Vujkovic-Cvijin et al., two host variables that appear to strongly affect the correlation between the gut microbiome and human diseases were identified: bowel movement quality and alcohol consumption (110). They proposed a profile of host variables; this profile serves as an insightful guide to minimize the “background” of individual differences in gut microorganisms and facilitate the identification of genuine disease states. Moreover, selecting specific time points for sample collection is crucial for minimizing the impact of circadian rhythm variations (111).

### 7.3 Fecal microbiota transplantation

Cutting-edge studies on fecal microbiota transplantation are offering valuable insights into disease management. Fecal microbial transplantation (FMT) has been utilized in various therapeutic applications to normalize the gut microbiota and improve physical function via the circulatory system to improve host metabolism, assist in immunomodulation, and address microecological imbalances caused by antibiotics, ultimately leading to positive outcomes (112, 113). Restoring a healthy gut microbial composition is a fundamental concept behind curing diseases.

The benefits of fecal bacterial transplantation further demonstrate the impact of the gut flora on extraintestinal organs. FMT, particularly with *Bifidobacterium*, can result in isoniazid-induced liver injury (23). However, it also confers benefits to overall health and reverses inflammation induced by specific bacteria, such as *Citrobacter*, in mice (77). This finding suggests a potential role for gut flora in modulating disease prognosis.

Microbial transplantation may not be suitable in all situations. In healthy individuals, there were no significant differences in the diversity and composition of gut microbiota before and after transplantation (112). Nonetheless, improvements in the gut microbiota were observed specifically in individuals with a low baseline microbial diversity (112). Low baseline microbiota diversity may partially reflect energy abnormalities without impairing the metabolic status. In the context of tuberculosis, it is important to consider the distinct benefits of short- and long-term microbial transplantation. In addition, autologous FMT has more benefits than heterologous FMT and may be a potential option in the future (114).

### 7.4 Microbial products

Maintaining the metabolic balance helps restore the microecology of the intestinal flora, thus preventing TB infection. Transplantation of more accurate and efficacious microbial components has been increasingly emphasized. Supplementation with *Lactobacillus* restores c-type lectin (mincle) expression in lung DCs, thereby enhancing immunity against *Mtb* (20). Moreover, *Lactobacillus casei* protect against anti-TB drug-induced liver injury by modulating the gut microflora and regulating intestinal inflammation via the TLR4–NF-κB–MyD88 pathways (115). In a tubercular mouse model, the production of palmitoleic acid by *A. muciniphila* strongly inhibited TB infection and was negatively correlated with the levels of tumor necrosis factors, such as TNF-α (47). Importantly, discontinuing anti-TNF therapy often leads to a paradoxical worsening of TB, particularly in patients with disseminated TB (116). However, oral administration of palmitoleic acid has been shown to remodel mucosal barriers and reduce inflammation by promoting the proliferation of anti-inflammatory bacteria such as *A. muciniphila* (117). The inverse relationship between palmitoleic acid and TNF suggests a molecular mechanism that differs from that of direct TNF blockade, implying that symbiotic bacterial products may offer alternative and gentler therapeutics to deliver anti-TB benefits to patients. Furthermore, specific gut microbiota may produce essential vitamins, such as B_6_, B_9_, and K, and also metabolize dietary compounds, such as flavonoids, which have been shown to have anti-*Mtb* effects (118, 119).

The use of probiotic and prebiotic products help reduce the duration and severity of inflammatory infections (120). Notably, gut bacterial metabolites, specifically those related to riboflavin, have been identified as potent stimulators of MAIT cells (58, 121–123). These T cells, which are imprinted by the microbiota, play a crucial role in immune development during early life and contribute significantly to the regulation of immune responses against pathogens. Butyrate, a beneficial SCFA, is currently under investigation as a potential therapeutic agent for its efficacy in the management of inflammatory diseases. Butyrate assists in regulating the integrity of the epithelial barrier and immune function, thereby facilitating interactions between macrophages and goblet cells (25). Reduced levels of butyrate, as evidenced by a significant reduction in the expression of the *bcoA* gene in the gut microbiota compared to that in healthy controls, have been linked to increased TB incidence (31, 124, 125). However, the accretion of acidic fermentation products, including SCFAs, reduces the luminal pH, leading to alterations in microbial composition and injury to the gut epithelium, with detrimental effects on health (126). These findings indicated that prebiotics may be counterproductive if not consumed in moderation.

## 8 Conclusion and perspectives

In conclusion, by elucidating the intricate interplay between gut microbiota and host immune systems, as well as their roles in modulating inflammation, metabolic pathways, and nutrient absorption, future research is poised to engineer more precise, personalized prevention strategies and treatment protocols. Several confounding factors, such as drug factors, living environment, lifestyle, immune status, and genetic variability, have been found to influence the diversity and composition of the gut microbiota (127). Targeting these factors can enhance human resistance against tuberculosis, thereby significantly curbing its incidence and dissemination.

Although investigating the gut microbiota as a potential adjunct to tuberculosis treatment is promising, high individual variation and the absence of a specific microbiota spectrum for TB pose significant challenges. These limitations preclude replacing traditional treatment approaches with gut microbiota-based therapies. Considering vaccine design, notably, researchers have uncovered a novel role of *Mycobacteria*, which may demonstrate protective effects and serve as strong non-specific immunopotentiators for the host (128). In the future, it may be possible to achieve tangible TB prevention through oral administration of specific beneficial microbes, thus leveraging the assistance of the gut microbiota. In addition to gut bacteria, the impact of fungi and viruses within the host intestinal tract on tuberculosis should not be ignored. Future research that integrates these analyses with other disease models will help us gain a more comprehensive understanding of the various factors that contribute to tuberculosis susceptibility.

Finally, we should be attentive to the delicate balance between maintaining the gut microbial equilibrium and combating tuberculosis with antibiotics to develop strategies that can effectively treat tuberculosis without disrupting the integrity of the gut microbiome. As our knowledge continues to expand, there is great potential to further improve our understanding of TB.

## 9 Method

The review was conducted in PubMed. The search strategy included the terms “gut microbiota,” “tuberculosis,” “lung,” “immunity,” and “antibiotic.” Studies were selected based on their relevance to the research question and publication date (within the last 10 years).
